# What moves patients to participate in prehabilitation before major surgery? A mixed methods systematic review

**DOI:** 10.1186/s12966-023-01474-6

**Published:** 2023-06-21

**Authors:** Miriam van der Velde, Marike van der Leeden, Edwin Geleijn, Cindy Veenhof, Karin Valkenet

**Affiliations:** 1 Research Group Innovation of Human Movement Care, Research Center for Healthy and Sustainable Living, HU University of Applied Sciences, Utrecht, NL Netherlands; 2 Department of Rehabilitation, Physical Therapy Science and Sports, University Medical Center Utrecht, Utrecht University, Utrecht, NL Netherlands; 3 Department of Rehabilitation Medicine, Amsterdam University Medical Center, Vrije Universiteit Amsterdam, Amsterdam, NL Netherlands; 4 Amsterdam Public Health Research Institute, Amsterdam University Medical Center, Vrije Universiteit Amsterdam, Amsterdam, NL Netherlands

**Keywords:** Prehabilitation, Behaviour change, Barriers, Facilitators

## Abstract

**Background:**

Prehabilitation offers patients the opportunity to actively participate in their perioperative care by preparing themselves for their upcoming surgery. Experiencing barriers may lead to non-participation, which can result in a reduced functional capacity, delayed post-operative recovery and higher healthcare costs. Insight in the barriers and facilitators to participation in prehabilitation can inform further development and implementation of prehabilitation. The aim of this review was to identify patient-experienced barriers and facilitators for participation in prehabilitation.

**Methods:**

For this mixed methods systematic review, articles were searched in PubMed, EMBASE and CINAHL. Articles were eligible for inclusion if they contained data on patient-reported barriers and facilitators to participation in prehabilitation in adults undergoing major surgery. Following database search, and title and abstract screening, full text articles were screened for eligibility and quality was assessed using the Mixed Method Appraisal Tool. Relevant data from the included studies were extracted, coded and categorized into themes, using an inductive approach. Based on these themes, the Capability, Opportunity, Motivation, Behaviour (COM-B) model was chosen to classify the identified themes.

**Results:**

Three quantitative, 14 qualitative and 6 mixed methods studies, published between 2007 and 2022, were included in this review. A multitude of factors were identified across the different COM-B components. Barriers included lack of knowledge of the benefits of prehabilitation and not prioritizing prehabilitation over other commitments (psychological capability), physical symptoms and comorbidities (physical capability), lack of time and limited financial capacity (physical opportunity), lack of social support (social opportunity), anxiety and stress (automatic motivation) and previous experiences and feeling too fit for prehabilitation (reflective motivation). Facilitators included knowledge of the benefits of prehabilitation (psychological capability), having access to resources (physical opportunity), social support and encouragement by a health care professional (social support), feeling a sense of control (automatic motivation) and beliefs in own abilities (reflective motivation).

**Conclusions:**

A large number of barriers and facilitators, influencing participation in prehabilitation, were found across all six COM-B components. To reach all patients and to tailor prehabilitation to the patient’s needs and preferences, it is important to take into account patients’ capability, opportunity and motivation.

**Trial registration:**

Registered in PROSPERO (CRD42021250273) on May 18th, 2021.

**Supplementary Information:**

The online version contains supplementary material available at 10.1186/s12966-023-01474-6.

## Background

Patients undergoing major surgery are at risk of adverse postoperative health outcomes such as complications and delayed or poor recovery [1, 2]. Prehabilitation offers patients the opportunity to actively participate in their perioperative care by preparing themselves for their upcoming surgery. By improving their functional capacity prior to surgery, patients enable themselves to better withstand the forthcoming stressor of major surgery, which can improve postoperative outcomes [1–4].

Early studies of interventions to improve functional capacity prior to surgery focused primarily on exercise training. Nowadays, the focus in prehabilitation is shifting towards a multimodal approach (i.e. exercise training, nutritional support, psychological support and/or coaching towards a healthy lifestyle). Current prehabilitation programs vary widely, including in terms of context (home-, community- or hospital-based), target population and degree of supervision [1–3, 5].

Although beneficial effects of prehabilitation have been shown, enrollment in prehabilitation programs remains challenging [6, 7]. Prehabilitation programs require active patient engagement. The patient’s choice whether or not to participate in prehabilitation can be influenced by the patients’ capability, opportunity and motivation [4, 8, 9]. Some may decline participation, which can result in a reduced functional capacity, delayed post-operative recovery and higher healthcare costs [1–4].

Insight into the reasons for (non-)participation in prehabilitation programs may be useful for health care professionals and researchers in the further development and implementation of prehabilitation interventions. Perceived barriers, such as the limited time frame prior to surgery and physical symptoms, may reduce or negatively affect participation in prehabilitation, while facilitators, such as social support and previous experiences with physical activity, may promote or positively affect participation [10]. A large body of evidence exists with both qualitative and quantitative (survey) data identifying barriers and facilitators to participation in prehabilitation in various surgical populations [6–8, 10–29]. The combination of qualitative and quantitative research can provide a more comprehensive analysis than each method alone and, therefore, a robust evidence base for further development and implementation of prehabilitation interventions. To our knowledge, no review has been published that systematically summarizes these barriers and facilitators. Therefore, the aim of this review was to identify patient-reported barriers and facilitators to participation in prehabilitation.

## Methods

### Design

A mixed methods systematic review was performed on attitudes towards participation in prehabilitation of people undergoing major surgery. This review was guided by the Joanna Briggs Institute (JBI) methodology for Mixed Methods Systematic Reviews [30] and the Preferred Reporting Items for Systematic Reviews and Meta Analyses (PRISMA) statement [31]. This review was registered at the International Prospective Register of Systematic Reviews (PROSPERO) database (CRD42021250273, May 18th, 2021).

### Search strategy

After scoping searches, three electronic databases (PubMed, EMBASE and CINAHL) were searched systematically to identify relevant articles up to 15 November 2022. The search strings were developed in collaboration with a research librarian.

The search strategy included keywords, synonyms, closely related words and index terms within the domains of the phenomena of interest, context and study type. The search strategy, including all identified keywords and index terms was adapted for each electronic database. Duplicate articles were excluded during the search strategy using the Amsterdam Efficient Deduplication (AED) method [32], and afterwards using RefWorks (ProQuest, Bethesda, MD). The complete search strategy for each database can be found in Additional file 1. In addition to the database searches, reference lists of the included full-text articles were screened to identify additional relevant articles.

### Inclusion and exclusion criteria

Articles were eligible for inclusion if they were published in English and available in full text. Articles had to contain qualitative and/or quantitative data on patient-reported barriers and facilitators to participation in prehabilitation in adults (≥ 18 years of age) undergoing major inpatient surgery.

Patient-reported factors are considered as barriers if they are described to negatively affect the patients choice to participate in prehabilitation. Factors are considered as facilitators if their presence is described to positively affect the patients choice to participate in prehabilitation.

For studies to be included, promoting physical activity or exercise had to be part of the prehabilitation intervention. Besides physical activity or exercise, prehabilitation could consist of interventions targeting other risk factors, such as smoking cessation or nutritional optimalisation. Exclusion criteria were theses, dissertations, editorials, research protocols and conference abstracts.

### Study selection

Following the search, all identified citations were collated and uploaded into Rayyan (Doha, Qatar). Titles and abstracts were then screened by two reviewers (MV and KV) independently using Rayyan for assessment against the in- and exclusion criteria. Studies that met the inclusion criteria were retrieved in full text and were screened by the two reviewers (MV and KV) independently. Any disagreements between the reviewers on eligibility were resolved through discussion.

### Assessment of methodological quality

Eligible studies were critically appraised for methodological quality using the Mixed Method Appraisal Tool (MMAT) by two reviewers (MV and KV) independently. This tool was developed for the appraisal of qualitative, quantitative and mixed methods studies and has been used in similar mixed method systematic reviews [33]. Any disagreements between the reviewers were resolved through discussion. As recommended, the MMAT was not used to calculate a score for individual studies, but was used to provide a context in which to interpret the findings. Studies were included in this review regardless of their methodological quality, to minimize study selection bias.

### Data extraction and analysis

A convergent integrated approach according to the JBI methodology for mixed methods systematic reviews was used in this study [30]. Study characteristics were extracted from the included studies by the primary author using a data extraction form. Data included author, year of publication, method (i.e. qualitative, quantitative, mixed methods), context, sample size, participant characteristics, phenomena of interest, data collection and data analysis.

To extract the findings relevant to the review objectives, studies were imported to ATLAS.ti version 22 (Berlin, Germany) for coding and analysis. Quantitative non-textual data were transformed into qualitized data to facilitate integration with data extracted from qualitative studies and the qualitative component of mixed methods studies. Qualitizing is the process of transforming quantitative data into qualitative data. This involves transformation of data into textual descriptions or narrative interpretation of quantitative results [30]. An inductive approach was taken by ‘free coding’ the findings of the studies using ATLAS.ti. Patient-reported barriers and facilitators were coded if they were described by the authors as influencing participants’ participation in prehabilitation. Then, these ‘free codes’ were organized into ‘themes’.

**Fig. 1:**
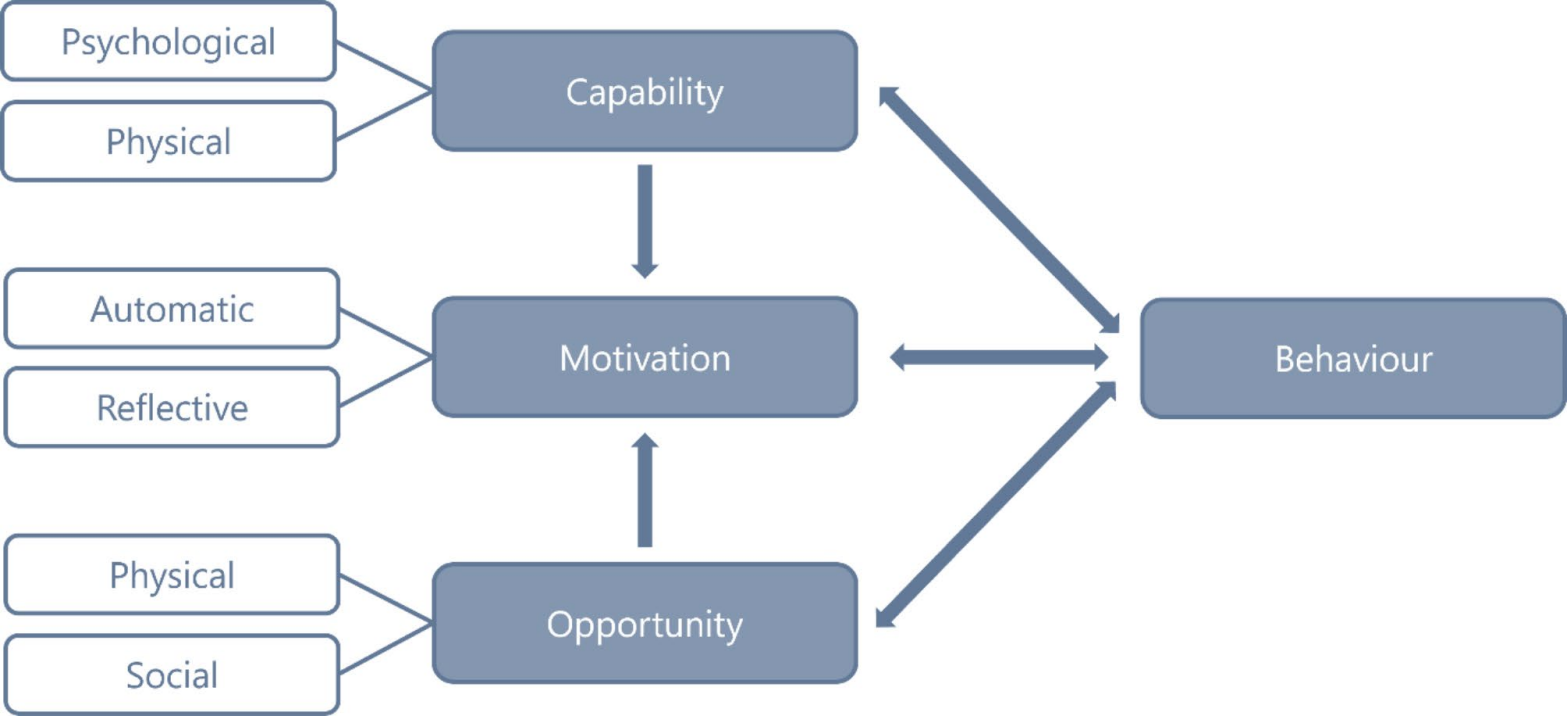
The COM-B system - a framework for understanding behaviour [9]

Based on these themes, the Capability, Opportunity, Motivation, Behaviour (COM-B) model (Fig. 1) was chosen to present the identified barriers and facilitators. The COM-B model is a model of behaviour and is part of the Behaviour Change Wheel, a theory-based framework for intervention development [9]. The COM-B model can be used on the level of an individual, group or population, and consists of six key components influencing behaviour: psychological capability, physical capability, social opportunity, physical opportunity, automatic motivation and reflective motivation [9]. Capability refers to a person’s psychological and physical ability to participate in an activity. Opportunity refers to external factors that make behaviour possible and motivation refers to the conscious and unconscious processes that direct and inspire behaviour [9]. In this study the COM-B model is used to explore perceived barriers and facilitators to identify potential levers for change to enhance participation in prehabilitation.

## Results

### Study inclusion

**Fig. 2:**
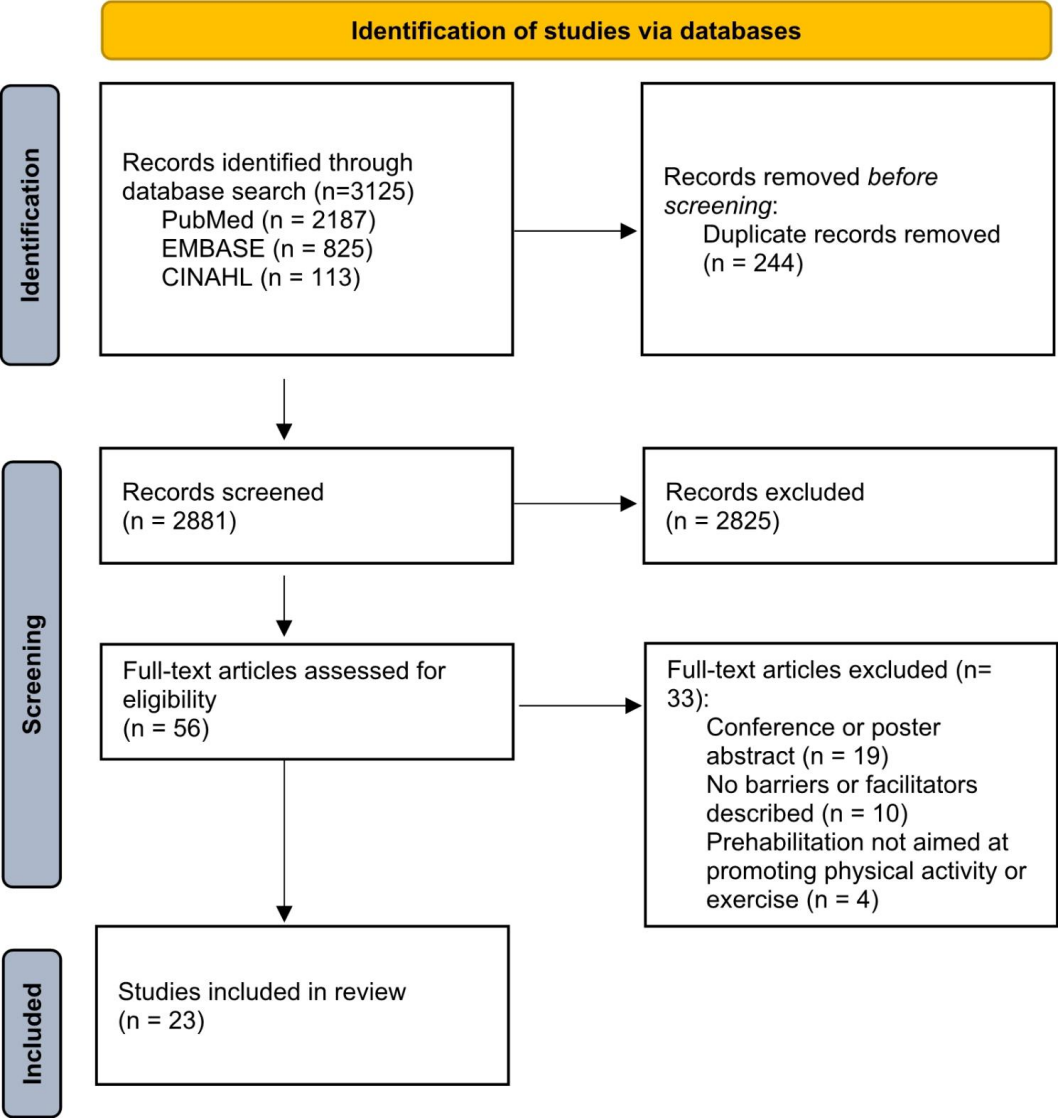
The PRISMA Flow Diagram of the literature search

The literature search generated 3125 studies: 2187 in PubMed, 825 in EMBASE, 113 in CINAHL. After removing duplicates, 2881 studies were included in the screening based on titles and abstracts. Of these articles, 56 were included for full-text assessment. Finally, of the 56 full-text articles, 23 articles were included in the review [6–8, 10–29]. The search and selection process is illustrated in Fig. 2.

### Characteristics of the studies

Three quantitative [6, 21, 25], 14 qualitative [7, 8, 11, 12, 15, 18–20, 23, 24, 26–29] and six mixed methods studies [10, 13, 14, 16, 17, 22], published between 2007 and 2022, were included in the review. Sample sizes varied between n = 7 and n = 103. Eighteen studies involved patients with cancer [6–8, 10–15, 17, 18, 20–24, 27, 29], two studies included patients with both malignant and non-malignant abdominal pathology [25, 26], one study involved patients awaiting total hip or knee replacement [16], one study involved patients with lumbar spine stenosis surgery [28] and one study involved patients awaiting coronary artery bypass surgery [19]. Further study characteristics can be found in Additional file 2.

### Methodological quality

The methodological quality of the included studies, assessed by means of the MMAT, varied widely. Sixteen studies satisfied all applicable MMAT quality criteria, indicating strong methodological quality [7, 8, 10–12, 15, 18–21, 23, 24, 26–29], the other seven studies did not fulfill all quality criteria or did not provide enough information to score all quality criteria [6, 13, 14, 16, 17, 22, 25].

All of the 14 qualitative studies had high methodological quality and provided a clear description of the research question, data collection and data analysis methods. Also, the results were supported adequately by qualitative data [7, 8, 11, 12, 15, 18–20, 23, 24, 26–29].

One of the three quantitative studies had a strong methodological quality [21]. Of the other two quantitative studies, one lacked information about the sampling strategy and nonresponse [25], while the other study did not pose a clear research question, making further appraisal impossible [6].

Of the six mixed methods studies, only one satisfied all MMAT criteria [10]. Aspects that resulted most commonly in a downgrade of quality for the mixed methods studies were: lack of information regarding participant recruitment rate, non-response, and lack of integration of qualitative and quantitative components.

A detailed presentation of the methodological quality per study is provided in Additional file 3.

### Findings of the review

The identified patient-reported barriers and facilitators per COM-B component and the corresponding articles reporting on these barriers and facilitators are presented in Table 1. A narrative of the identified barriers and facilitators to participation in prehabilitation is provided per COM-B component. Barriers and facilitators are presented regardless of the number of studies in which they were described as the frequency of reporting is primarily due to the design and methods used and cannot be used as an indicator of importance [34].

**Table 1:** Barriers and facilitators to participation in prehabilitation in people undergoing major surgery

COM-B Component	Barriers	Facilitators
Psychological capability	Lack of knowledge of the benefits and concept of prehabilitation [8, 18, 26, 28], no awareness of prehabilitation options [27], no priority due to other commitments [8, 13, 16, 22], lack of technological skills [21]	Knowledge of the benefits of prehabilitation [12, 19]
Physical capability	Physical symptoms [7, 8, 10, 11, 13, 15–18, 20–23, 27, 28], age-related limitations [18], physical and exercise limitations [11, 18, 23, 25, 28], comorbidities [11, 18]	
Physical opportunity	Hard to find time [6, 8, 10, 14, 18, 20, 21, 27], limited financial capacity [20, 21, 28], transportation and parking limitations [6, 10, 12, 14, 18, 20, 21, 23–25, 27, 28], lack of physical activity resources [18], short pre-operative timeframe [8, 11, 18, 22, 23, 25, 26], bad weather [7, 10, 13, 14, 17], hospital appointments [10, 11, 14, 18], living alone [23], work [8, 13, 16, 20], too general recommendations [13]	Having access to physical activity resources [10], home-based prehabilitation [8, 14, 27], neighborhood walkability [10], tailored approach [11, 13, 22, 24, 26, 27, 29], resources for registration of activities and monitoring of exercise intensity [8, 10, 12–14, 22]
Social opportunity	Lack of social support [10], contact with fellow patients [7, 11]	Social support [6–8, 10–13, 18, 19], encouragement by family and friends [7, 8, 10, 12, 13, 18, 23, 27], being part of a peer group [6, 11, 12, 16, 19, 23, 24], exercise prescription by the doctor [6, 12, 17, 21, 27, 29], collaborating with the health care professional [7, 22], encouragement by a health care professional [6, 8, 10–13, 18–23], supervision [12, 14, 22]
Automatic motivation	Anxiety [8, 13, 14, 21, 27], emotional impact of cancer diagnosis [11, 18], state of mind [8, 10, 18, 20, 21, 27], stress [23]	Measures of progression [7, 12], positive distraction [7, 14, 18, 22], sense of control [7, 8, 12–14, 26], sense of purpose [7, 8, 12, 13, 18], emotional impact of cancer diagnosis [27], positive mindset [27]
Reflective motivation	Lack of self-confidence [8, 18, 21], lack of willpower [8, 18], previous positive experiences with postoperative recovery [18], previous negative experiences with exercise [18], doubting effectiveness [18], lack of motivation [14, 21, 25], feeling too fit for prehabilitation [8, 11, 18, 21, 23, 24], unattainable goals [7]	High belief in own abilities [18], previous positive experiences with exercise [10–12, 18, 21], anticipating better postoperative recovery [7, 8, 10, 12, 14, 18, 20, 22–24, 27–29], anticipating physical improvements [6–8, 12, 20, 22, 24, 27, 29], collaboration and mutual goal setting [7, 8, 27], motivation [10, 23]

### Psychological capability

Within the domain of psychological capability both barriers and facilitators to participation in prehabilitation were identified [8, 10, 12–14, 16–19, 21, 22, 26–28]. Knowledge of the benefits of prehabilitation was a facilitator [12, 19]. Conversely, a lack of knowledge of the concept and benefits of prehabilitation, and the patients’ perception that it is important to conserve energy for the recovery after surgery, were barriers to participation in prehabilitation [8, 18, 26, 28]. Also, the lack of patient awareness of the options regarding prehabilitation was reported as a barrier [27]. Patients who did not prioritize prehabilitation in the run-up to their surgery, were less likely to participate in prehabilitation. Patients prioritized for example spending time with their family and living their everyday lives over prehabilitation [8, 13, 16, 22]. One article reported the use of technology/telehealth as part of the intervention to be a barrier to participation in prehabilitation as this requires technological skills the patient could be lacking [21].

### Physical capability

Feeling physically incapable was identified as an important barrier to participation in prehabilitation, within the domain of physical capability [7, 8, 10, 11, 13, 15–18, 20–23, 25, 27, 28]. Experiencing physical symptoms such as fatigue, nausea or pain, in some cases directly related to (cancer) treatment, contributes greatly to the feeling of physical incapability [7, 8, 10, 11, 13, 15–18, 20–23, 27, 28]. Also, experiencing age-related or exercise limitations, and the presence of comorbidities, such as joint pain by osteoarthritis, were reported as a limiting factors in the physical capability of patients preparing for major surgery [11, 18, 23, 25, 28].

### Physical opportunity

The availability of time, finances, good weather and resources were identified as facilitators of participation in prehabilitation when available, and as a barrier when lacking [6, 8, 10, 14, 18, 20, 21, 27]. Patients reported finding it hard to fit in prehabilitation with their work, medical appointments and practical tasks like taking care of the household [8, 10, 11, 13, 14, 16, 18, 20]. This was especially the case with facility-based prehabilitation. Home-based prehabilitation was reported to be a more practical solution, as patients could fit it in around work and other activities [8, 14, 27]. Home-based prehabilitation also helped overcome the barriers of transportation and parking, which were frequently reported by patients in the case of facility-based prehabilitation [6, 10, 12, 14, 18, 20, 21, 23–25, 27, 28]. Patients addressed the importance of a tailored approach, based on individual needs and preferences. Too general recommendations could represent as a barrier to participation in prehabilitation, as patients may see the advice as not important or appropriate to them [11, 13, 22, 24, 26, 27, 29]. Besides finding time in relation to other tasks and activities, the short preoperative period was also perceived as a barrier, leaving little time for prehabilitation [8, 11, 18, 22, 23, 25, 26]. Limited financial capacity and the absence of physical activity resources were reported as barriers to participation, while having access to physical activity resources facilitated participation in prehabilitation [10, 18, 20, 21, 28]. The ability to monitor activity, as part of the prehabilitation intervention, was identified as a motivational factor in some patients. These patients experienced keeping an exercise log or ‘ticking boxes’ for performed activities as facilitators to participation in prehabilitation [8, 10, 12–14, 22].

### Social opportunity

Within the domain of social opportunity, social and peer support were widely reported as facilitators of participation in prehabilitation, whereas a lack of social support was reported as a barrier [6–8, 10–13, 18, 19]. Encouragement and support from family and friends were important motivational factors [7, 8, 10, 12, 13, 18, 23, 27]. Also, the interaction with other patients when attending a prehabilitation program was mentioned as a facilitator to participate in prehabilitation. Many patients enjoyed having company during exercise, sharing experiences and supporting each other, but for some the experiences with other patients could also be a barrier to participating in a prehabilitation program [6, 7, 11, 12, 16, 19, 23, 24]. The role of health care professionals was identified as very important [6–8, 10–14, 17–23, 27, 29]. The recommendation from a health care professional to participate in prehabilitation was an important facilitator for patients, especially when it was recommended by their medical doctor [6, 12, 17, 21, 27, 29]. The support and encouragement by health care professionals, engaging in a collaboration with the health care professionals, supervision and regular contact were all identified as facilitators to participation in prehabilitation [6–8, 10–14, 17–23].

### Automatic motivation

The emotional wellbeing of patients was identified as an important factor within the domain of automatic motivation [8, 10, 11, 13, 14, 18, 20–23, 27]. The impact of the (cancer) diagnosis comes with feelings of insecurity, anxiety, stress and a fear of exercise. These emotions can hinder patients to participate in prehabilitation [8, 10, 11, 13, 14, 18, 20, 21, 23] but can also enhance the motivation to change to ‘beat the disease’ [27]. Some patients reported that engaging in prehabilitation during the stressful preoperative period provided a positive distraction, reduced sad thoughts and created meaning. Participation in prehabilitation can help patients to regain a sense of control and purpose by making the best use of time available before surgery [7, 8, 12–14, 18, 22, 26]. Reinforcement, by objective measures of progression, increases motivation and has a positive influence on exercise adherence [7, 12].

### Reflective motivation

Negative beliefs about capabilities and negative attitudes towards prehabilitation and exercise in general were reported as important barriers to participation in prehabilitation [8, 11, 12, 18, 21, 23, 24]. Lacking self-confidence and willpower were identified barriers to participation. Given the nature of their medical situation some patients found it difficult to find the energy to participate in prehabilitation or to make lifestyle changes. [8, 18, 21]. Previous experiences with exercise or surgery could act as both barriers and facilitators. If postoperative recovery was a positive experience, patients felt ready for surgery without additional effort, despite changes in context and health status, whereas negative experiences with postoperative recovery could motivate patients to actively engage in prehabilitation [18]. Previously experienced benefits of exercise were identified as facilitators [10–12, 18, 21], while previous negative experiences with exercise and doubting the effectiveness of prehabilitation act as barriers [18]. Some patients did not see the need to participate in prehabilitation as they believed themselves to be too fit or sufficiently active [8, 11, 18, 21, 23, 24] and others lacked motivation or interest in exercise [14, 21, 25]. The belief that prehabilitation enhances recovery after surgery [7, 8, 10, 12, 14, 18, 20, 22–24, 27–29] and can lead to physical and mental improvements was an important factor motivating patients to participate in prehabilitation [6–8, 12, 20, 22, 24, 27, 29]. Patients expressed the desire to ‘play their part’ in their surgical journey by improving their fitness before surgery [7]. Also, goal setting was mentioned as an important factor to ensure adherence, but some patients mentioned that setting goals too high can be demotivating and can lead to patients giving up [7, 8, 27].

## Discussion

To our knowledge, this is the first mixed-methods review on patient-reported barriers and facilitators to participation in prehabilitation in patients undergoing major surgery. The identified barriers and facilitators were multidimensional and suggest that participation in prehabilitation is affected by the patients’ capability, opportunity and motivation, reflecting the need for personalized approach regarding prehabilitation.

Prehabilitation programs require significant patient involvement. Patients must decide whether to participate in prehabilitation and commit to (multifactorial) short term behaviour change such as increasing physical activity or smoking cessation prior to surgery [4, 35]. Many models, theories and frameworks exist to understand behaviour and design interventions to bring about behaviour change [9]. The COM-B model describes that behaviour is part of a system that includes a patient’s capability to perform a behaviour, and the opportunity and motivation to carry out that behaviour [9].

Within the domain of psychological capability, we identified both barriers and facilitators [7, 8, 10, 11, 13, 15–18, 20–23, 25, 27, 28]. Having the necessary knowledge about the benefits of prehabilitation influences participation in prehabilitation [8, 12, 18, 19, 26, 28]. A lack of information and advice from health care professionals may lead to negative beliefs about prehabilitation, which can hinder participation. In the field of cardiac rehabilitation, similar results have been reported. Physician recommendation is considered very important in the decision to participate in cardiac rehabilitation, especially in patients ambivalent about participating [36]. Therefore, health care professionals, such as physicians, nurse practitioners and physical therapists should actively inform and advise patients about the benefits of prehabilitation to help people overcome barriers [37]. That being said, timing of the information is of the utmost importance, as the shock of diagnosis and information overload could prevent information from being remembered by patients [36].

Within the domain of physical capability, only barriers were reported. These barriers are consistent with barriers reported in studies of barriers and facilitators to physical activity and exercise in general, in diverse populations [38–40]. Feeling physically incapable due to physical symptoms, comorbidities or limited fitness hindered participation in prehabilitation [7, 8, 10, 11, 13, 15–18, 20–23, 25, 27, 28]. In patients with osteoarthritis for example, pain and stiffness are frequently reported barriers to exercise, even though physical exercise has been shown to have a positive effect on these factors [41]. Support and education from health care professionals and tailoring prehabilitation to the patients abilities and needs can help overcome the physical barriers to participation in prehabilitation.

Identified factors concerning the physical opportunity were, among others, availability of time and resources and travel limitations. Patients find it difficult to make time for prehabilitation and fit it in around other tasks, and are reluctant to travel to attend prehabilitation [6, 8, 10–14, 16, 18, 20, 21, 23–28]. Therefore, home- or community based prehabilitation, or prehabilitation supported by eHealth might be considered for these patients as they are more easily incorporated in one’s daily life. Also, high-risk patients are often elderly who may be dependent on others for travelling, making home- or community based prehabilitation more accessible [42, 43].

Within the domain of social opportunity, peer support and support of a health care professional were found to be very important. These findings are similar to studies of barriers and facilitators to physical activity in other populations [38–41]. Also, studies providing supervision are more likely to find a beneficial effect of prehabilitation on postoperative outcomes, which highlights the importance of health care professionals involvement in prehabilitation [5]. Providing home- or community based prehabilitation could stimulate involvement of caregivers and social support.

Within the domain of automatic motivation, we found the emotional wellbeing of the patient to be an important barrier [8, 10, 11, 13, 14, 18, 20, 21, 23, 27]. This is in accordance with literature on patients with coronary heart disease. The loss of confidence, shock of diagnosis and life stress impacted participation in cardiac rehabilitation [36]. Within the domain of reflective motivation the patient’s intentions and beliefs play an important role in participation in prehabilitation [7, 8, 10–14, 18, 20–27, 29]. The preoperative period is often considered a teachable moment: a time when patients may be more receptive to changing their risk behaviour. It is described that, for example, receiving a cancer diagnosis can increase the patient’s motivation to change risk behaviour; people see a need for change, in the light of their upcoming surgery [4]. Prehabilitation interventions can make use of this teachable moment, by focusing on short term life style changes in the preoperative period as patients demonstrate greater motivation, confidence and higher prioritization around behaviour change for peri-operative benefits compared to long term health benefits. This pre-operative motivation has the potential to be utilized to encourage a long(er)-term behaviour change [3, 4, 44]. Offering prehabilitation at home or in the community could make it easier for patients to follow through on the behaviour change after surgery, as they acquired skills to change behaviour in their own environment.

While all barriers and facilitators could be categorized within the components of the COM-B model, the identified factors are interrelated, which is in line with the hypothesized relationships between the four components of the COM-B model [9]. For example, we found that patients who felt ill or experienced physical symptoms (physical capability) had less confidence in their own abilities (reflective motivation). Also, patient’s perceived need for participation (reflective motivation) was influenced by the knowledge of the benefits of prehabilitation (psychological capability). Capability and opportunity influence the relationship between motivation and behaviour, rather than the behaviour itself. So, by changing capabilities and opportunities, we can influence a person’s motivation and therefore encourage behaviour change. These interactions should be taken into account when considering the barriers and facilitators for participation in prehabilitation.

In this review we included all studies regarding patients undergoing major inpatient surgery, regardless of their medical diagnosis. Nevertheless, the majority of studies involved patients with cancer or abdominal pathology. Despite the fact that only a small amount of studies involved patients with other medical diagnoses, the identified barriers and facilitators are consistent across studies.

The results regarding the effectiveness of prehabilitation are promising but not conclusive. Also, there is no consensus on the optimal approach to delivering prehabilitation [1–3, 5]. Supervised face-to-face programs delivered by health care professionals are considered the gold standard [1–3, 5]. But, as this review shows, there is also evidence that patients face barriers to attending these prehabilitation programs. And while some patients prefer home-based programs with or without eHealth support, others express the need for peer-support and involvement from health care professionals, highlighting the need for personalized prehabilitation.

With an understanding of the capability, opportunity and motivational barriers and facilitators, health care professionals and researchers can work through the steps of the Behaviour Change Wheel. The Behaviour Change Wheel is a framework designed to aid intervention designers in moving from a behavioral analysis of a problem to an evidence-based intervention method [9]. It can be used to identify intervention functions, behaviour change techniques and implementation strategies to bring about change. The presented overview of factors influencing participation in prehabilitation can be used as a starting-point in developing, evaluating and/or implementing prehabilitation interventions.

### Strengths and limitations

First, a strength of this review was the mixed-methods approach that allowed the synthesis of findings from quantitative, qualitative and mixed-methods studies, providing a rich set of data. Second, this study adopted an inductive approach to data analysis. The primary purpose of an inductive approach is to allow research findings to emerge from the data. This ensured that the researchers assessed barriers and facilitators with an open mind and from a broad perspective without the restrictions of a pre-selected framework. The final choice for the COM-B model, as a means for presenting the results, was made based on the results of the inductive analysis.

We also recognize some limitations in this study. First, in this review, we included all studies irrespective of their methodological quality scores, which may have resulted in the inclusion of low-quality evidence. However, this was done to minimize the risk of study selection bias. Second, we followed the recommendation of the MMAT authors to not calculate an overall total quality score [33]. This can make it hard to compare methodological quality between studies and value the results of the studies in the light of study quality. However, with this we followed the recommendations of the JBI guideline for mixed methods systematic reviews to not perform an assessment of the certainty of evidence due to the complexity of combining both quantitative and qualitative research [30]. Third, most studies included in this review focused on patients who completed prehabilitation. Only a few studies focused on the attitudes of patients who were not (yet) exposed to prehabilitation. So, information from patients not receiving or declining prehabilitation is limited.

## Conclusions

This review provides a comprehensive overview of patient-reported barriers and facilitators to participation in prehabilitation prior to major surgery. Understanding the capability, opportunity and motivational barriers and facilitators of the COM-B model can be used as a starting point for designing or improving interventions. Given the wide and extensive range of barriers and facilitators that influence participation in prehabilitation a single solution is unlikely. The large number of barriers and facilitators described in the various COM-B components reflect the individual differences and the need for a personalized approach. Within prehabilitation, we need to develop solutions that are flexible, adaptable to the patients’ needs and can be provided in different contexts.

## Electronic supplementary material

Below is the link to the electronic supplementary material.

Additional file 1: Search strategy per database

Additional file 2: Characteristics of included studies

Additional file 3: Methodological quality per study according to the MMAT-criteria

## Data Availability

Not applicable.

## Abbreviations

AED: Amsterdam Efficient Deduplication
JBI: Joanna Briggs Institute
MMAT: Mixed Method Appraisal Tool
COM-B: Capability, Opportunity, Motivation, Behaviour
