# Supplementary material for: What moves patients to participate in prehabilitation before major surgery? A mixed methods systematic review
Source: Int J Behav Nutr Phys Act. 2023 Jun 21;20:75. doi: 10.1186/s12966-023-01474-6 (PMC10286498; doi:10.1186/s12966-023-01474-6)
Supplement: Supplementary file 1 — Additional file 1: Search strategy per database [file 12966_2023_1474_MOESM1_ESM.docx]

**Additional file 1 Search strategy per database**

PubMed

1. Phenomena of interest (532088 hits)

barrier*[Title/Abstract] OR challenge*[Title/Abstract] OR issue*[Title/Abstract] OR difficult*[Title/Abstract] OR facilitator*[Title/Abstract] OR enabler*[Title/Abstract] OR "Motivation"[Mesh] OR motivat*[Title/Abstract] OR belief*[Title/Abstract] OR preference*[Title/Abstract] OR view*[Title/Abstract] OR opinion*[Title/Abstract] OR "Attitude to Health"[Mesh] OR attitude*[Title/Abstract] OR perception*[Title/Abstract] OR experience*[Title/Abstract] OR thought*[Title/Abstract] OR feeling*[Title/Abstract] OR perspective*[Title/Abstract]

2. Context (74617 hits)

prehabilitat*[Title/Abstract] OR pre-habilitat*[Title/Abstract] OR "Preoperative Exercise"[MeSH Terms] OR "preoperative exercise*"[Title/Abstract] OR "pre-operative exercise*"[Title/Abstract] OR "preoperative rehabilitation"[Title/Abstract] OR "pre-operative rehabilitation"[Title/Abstract] OR "preoperative physical therapy"[Title/Abstract] OR "pre-operative physical therapy"[Title/Abstract] OR "preoperative physiotherapy"[Title/Abstract] OR "pre-operative physiotherapy"[Title/Abstract] OR "Preoperative Care"[MeSH Terms] OR "preoperative care"[Title/Abstract] OR "pre-operative care"[Title/Abstract] OR "preoperative program*"[Title/Abstract] OR "pre-operative program*"[Title/Abstract] OR "preoperative intervention*"[Title/Abstract] OR "pre-operative intervention*"[Title/Abstract]

3. Method of data collection (2447069 hits)

"Qualitative Research"[Mesh] OR qualitative[Title/Abstract] OR "Focus Groups"[MeSH Terms] OR "focus group*"[Title/Abstract] OR "Interviews as Topic"[MeSH Terms] OR interview*[Title/Abstract] OR "Surveys and Questionnaires"[MeSH Terms] OR survey*[Title/Abstract] OR questionnaire*[Title/Abstract] OR phenomenolog*[Title/Abstract] OR "Grounded Theory"[Mesh] OR "grounded theory"[Title/Abstract] OR "mixed method*"[Title/Abstract] OR "mixedmethod*"[Title/Abstract] OR "multi method*"[Title/Abstract] OR "multimethod*"[Title/Abstract]

4. Combined (2187 hits)

1 AND 2 AND 3

Embase

1. Phenomena of interest (6542294 hits)

barrier*:ti,ab,kw OR challenge*:ti,ab,kw OR issue*:ti,ab,kw OR difficult*:ti,ab,kw OR facilitator*:ti,ab,kw OR enabler*:ti,ab,kw OR 'motivation'/exp OR motivat*:ti,ab,kw OR belief*:ti,ab,kw OR preference*:ti,ab,kw OR view*:ti,ab,kw OR opinion*:ti,ab,kw OR 'patient attitude'/exp OR attitude*:ti,ab,kw OR perception*:ti,ab,kw OR experience*:ti,ab,kw OR thought*:ti,ab,kw OR feeling*:ti,ab,kw OR perspective*ti,ab,kw

2. Context (53660 hits)

‘prehabilitat*’:ti,ab,kw OR ‘pre-habilitat*’:ti,ab,kw OR 'preoperative exercise'/exp OR ‘preoperative exercise*’:ti,ab,kw OR ‘pre-operative exercise*’:ti,ab,kw OR ‘preoperative rehabilitation’:ti,ab,kw OR ‘pre-operative rehabilitation’:ti,ab,kw OR ‘preoperative physical therapy’:ti,ab,kw OR ‘pre-operative physical therapy’:ti,ab,kw OR 'preoperative physiotherapy':ti,ab,kw OR ‘pre-operative physiotherapy’:ti,ab,kw OR 'preoperative care'/exp OR ‘preoperative care’:ti,ab,kw OR ‘pre-operative care’:ti,ab,kw OR ‘preoperative program*’:ti,ab,kw OR ‘pre-operative program*’:ti,ab,kw OR ‘preoperative intervention*’:ti,ab,kw OR ‘pre-operative intervention*’:ti,ab,kw

3. Method of data collection (2573620 hits)

'qualitative research'/exp OR 'qualitative':ti,ab,kw OR 'focus group*':ti,ab,kw OR 'interview'/exp OR 'interview*':ti,ab,kw OR 'questionnaire'/exp OR 'survey*':ti,ab,kw OR 'questionnaire*':ti,ab,kw OR 'phenomenology'/exp OR 'phenomenolog*':ti,ab,kw OR 'grounded theory'/exp OR 'grounded theory':ti,ab,kw OR 'mixed method*':ti,ab,kw OR 'mixedmethod*':ti,ab,kw OR 'multi method*':ti,ab,kw OR 'multimethod*':ti,ab,kw

4. Combined (1498 hits)

1 AND 2 AND 3

5. Articles from Pubmed search excluded (825 hits)

Cinahl

1. Phenomena of interest (1623118 hits)

MH "Patient Attitudes" OR TI barrier* OR AB barrier* OR TI challenge* OR AB challenge* OR TI issue* OR AB issue* OR TI difficult* OR AB difficult* OR TI facilitator* OR AB facilitator* OR TI enabler* OR AB enabler* OR TI motivat* OR AB motivat* OR TI belief* OR AB belief* OR TI preference* OR AB preference* OR TI view* OR AB view* OR TI opinion* OR AB opinion* OR TI attitude* OR AB attitude* OR TI perception* OR AB perception* OR TI experience* OR AB experience* OR TI thought* OR AB thought* OR TI feeling* OR AB feeling* OR TI perspective* OR AB perspective*

2. Context (1320 hits)

MH prehabilitation OR TI prehabilitat* OR AB prehabilitat* OR TI pre-habilitat* OR AB pre-habilitat* OR TI “preoperative exercise*” OR AB “preoperative exercise*” OR TI “pre-operative exercise*” OR AB “pre-operative exercise*” OR TI "preoperative rehabilitation" OR AB "preoperative rehabilitation" OR TI "pre-operative rehabilitation" OR AB "pre-operative rehabilitation" OR TI "preoperative physical therapy" OR AB "preoperative physical therapy" OR TI "pre-operative physical therapy" OR AB "pre-operative physical therapy" OR TI "preoperative physiotherapy" OR AB "preoperative physiotherapy" OR TI "pre-operative physiotherapy" OR AB "pre-operative physiotherapy" OR TI "preoperative care" OR AB "preoperative care" OR TI "pre-operative care" OR AB "pre-operative care" OR TI "preoperative program*" OR AB "preoperative program*" OR TI "pre-operative program*" OR AB "pre-operative program*" OR TI "preoperative intervention*" OR AB "preoperative intervention*" OR TI "pre-operative intervention*" OR AB "pre-operative intervention*"

3. Method of data collection (950894 hits)

MH "Qualitative studies" OR TI Qualitative OR AB Qualitative OR MH "Focus Groups" OR TI “Focus Group*” OR AB “Focus Group*” OR MH "Interviews" OR TI "interview*" OR AB “interview*” OR MH "Surveys" OR TI "survey*" OR AB "survey*" OR TI "questionnaire*" OR AB "questionnaire*" OR TI phenomenolog* OR AB phenomenolog* OR MH "Grounded Theory" OR TI "grounded theory" OR AB "grounded theory" OR MH "Multimethod Studies" OR TI "mixed method*" OR AB "mixed method*" OR TI "mixedmethod*" OR AB "mixedmethod*" OR TI "multi method*" OR AB "multi method*" OR TI "multimethod*" OR AB "multimethod*"

4. Combined (113 hits)

1 AND 2 AND 3
