# Supplementary material for: What moves patients to participate in prehabilitation before major surgery? A mixed methods systematic review
Source: Int J Behav Nutr Phys Act. 2023 Jun 21;20:75. doi: 10.1186/s12966-023-01474-6 (PMC10286498; doi:10.1186/s12966-023-01474-6)
Supplement: Supplementary file 2 — Additional file 2: Characteristics of included studies [file 12966_2023_1474_MOESM2_ESM.docx]

**Additional file 3 Methodological quality per study according to the MMAT-criteria**

|  |  | **Screening questions** | | **Qualitative studies** | | | | | **Randomized Controlled Trials** | | | | | **Non-Randomized studies** | | | | | **Quantitative descriptive studies** | | | | | **Mixed Methods studies** | | | | |
| --- | --- | --- | --- | --- | --- | --- | --- | --- | --- | --- | --- | --- | --- | --- | --- | --- | --- | --- | --- | --- | --- | --- | --- | --- | --- | --- | --- | --- |
|  | **Type of study** | **S1** | **S2** | **1.1** | **1.2** | **1.3** | **1.4** | **1.5** | **2.1** | **2.2** | **2.3** | **2.4** | **2.5** | **3.1** | **3.2** | **3.3** | **3.4** | **3.5** | **4.1** | **4.2** | **4.3** | **4.4** | **4.5** | **5.1** | **5.2** | **5.3** | **5.4** | **5.5** |
| Agasi-Idenburg et al, 2020 [1] | Qualitative | Y | Y | Y | Y | Y | Y | Y |  |  |  |  |  |  |  |  |  |  |  |  |  |  |  |  |  |  |  |  |
| Banerjee et al, 2021 [2] | Qualitative | Y | Y | Y | Y | Y | Y | Y |  |  |  |  |  |  |  |  |  |  |  |  |  |  |  |  |  |  |  |  |
| Beck at al, 2020 [3] | Qualitative | Y | Y | Y | Y | Y | Y | Y |  |  |  |  |  |  |  |  |  |  |  |  |  |  |  |  |  |  |  |  |
| Beck et al, 2021 [4] | Mixed Methods | Y | Y | Y | Y | Y | Y | Y |  |  |  |  |  |  |  |  |  |  | Y | Y | Y | C | Y | Y | Y | Y | Y | Y |
| Beck et al, 2021 [5] | Mixed Methods | Y | Y | Y | Y | Y | Y | Y |  |  |  |  |  |  |  |  |  |  | C | C | Y | C | C | N | N | N | C | N |
| Brahmbhatt et al, 2020 [6] | Mixed Methods | Y | Y | Y | Y | Y | Y | Y |  |  |  |  |  | Y | Y | N | N | Y |  |  |  |  |  | Y | Y | Y | Y | Y |
| Burke et al, 2013 [7] | Qualitative | Y | Y | Y | Y | Y | Y | Y |  |  |  |  |  |  |  |  |  |  |  |  |  |  |  |  |  |  |  |  |
| Clode et al, 2018 [8] | Mixed Methods | Y | Y | Y | Y | Y | Y | Y |  |  |  |  |  | Y | Y | N | N | Y |  |  |  |  |  | Y | N | N | Y | Y |
| Collaço et al, 2021 [9] | Qualitative | Y | Y | Y | Y | Y | Y | Y |  |  |  |  |  |  |  |  |  |  |  |  |  |  |  |  |  |  |  |  |
| Cooper et al, 2022 [10] | Qualitative | Y | Y | Y | Y | Y | Y | Y |  |  |  |  |  |  |  |  |  |  |  |  |  |  |  |  |  |  |  |  |
| Daun et al, 2022 [11] | Qualitative | Y | Y | Y | Y | Y | Y | Y |  |  |  |  |  |  |  |  |  |  |  |  |  |  |  |  |  |  |  |  |
| Ferreira et al, 2018 [12] | Quantitative | N | C |  |  |  |  |  |  |  |  |  |  |  |  |  |  |  | C | Y | Y | C | Y |  |  |  |  |  |
| Finley et al, 2020 [13] | Mixed Methods | Y | Y | Y | C | Y | N | Y |  |  |  |  |  |  |  |  |  |  | Y | Y | Y | N | Y | Y | Y | N | Y | N |
| Gurunathan et al, 2022 [14] | Quantitative | Y | Y |  |  |  |  |  |  |  |  |  |  |  |  |  |  |  | C | C | Y | C | Y |  |  |  |  |  |
| Karlsson et al, 2020 [15] | Qualitative | Y | Y | Y | Y | Y | Y | Y |  |  |  |  |  |  |  |  |  |  |  |  |  |  |  |  |  |  |  |  |
| Lam et al, 2022 [16] | Qualitative | Y | Y | Y | Y | Y | Y | Y |  |  |  |  |  |  |  |  |  |  |  |  |  |  |  |  |  |  |  |  |
| Mooney et al, 2007 [17] | Qualitative | Y | Y | Y | Y | Y | Y | Y |  |  |  |  |  |  |  |  |  |  |  |  |  |  |  |  |  |  |  |  |
| Parker et al, 2019 [18] | Mixed Methods | Y | Y | Y | Y | Y | Y | Y |  |  |  |  |  |  |  |  |  |  | Y | Y | Y | Y | Y | Y | Y | Y | Y | Y |
| Polen-De et al, 2021 [19] | Qualitative | Y | Y | Y | Y | Y | Y | Y |  |  |  |  |  |  |  |  |  |  |  |  |  |  |  |  |  |  |  |  |
| van der Zanden et al, 2021 [20] | Qualitative | Y | Y | Y | Y | Y | Y | Y |  |  |  |  |  |  |  |  |  |  |  |  |  |  |  |  |  |  |  |  |
| Wang et al, 2022 [21] | Qualitative | Y | Y | Y | Y | Y | Y | Y |  |  |  |  |  |  |  |  |  |  |  |  |  |  |  |  |  |  |  |  |
| Waterland et al, 2021 [22] | Quantitative | Y | Y |  |  |  |  |  |  |  |  |  |  |  |  |  |  |  | Y | Y | Y | Y | Y |  |  |  |  |  |
| Wu et al, 2022 [23] | Qualitative | Y | Y | Y | Y | Y | Y | Y |  |  |  |  |  |  |  |  |  |  |  |  |  |  |  |  |  |  |  |  |

Abbreviations: Y = yes; N = no, C = can’t tell

**References**

1. Agasi-Idenburg CS, Zuilen MK, Westerman MJ, Punt CJA, Aaronson NK, Stuiver MM. I am busy surviving - Views about physical exercise in older adults scheduled for colorectal cancer surgery. Journal of geriatric oncology 2020(11):444-450.

2. Banerjee S, Semper K, Skarparis K, Naisby J, Lewis L, Cucato G, Mills R, Rochester M, Saxton J. Patient perspectives of vigorous intensity aerobic interval exercise prehabilitation prior to radical cystectomy: a qualitative focus group study. Disabil Rehabil 2021(43):1084-1091.

3. Beck A, Thaysen HV, Soegaard CH, Blaakaer J, Seibaek L. Investigating the experiences, thoughts, and feelings underlying and influencing prehabilitation among cancer patients: a qualitative perspective on the what, when, where, who, and why. Disabil Rehabil 2020:1-8.

4. Beck A, Thaysen HV, Soegaard CH, Blaakaer J, Seibaek L. Prehabilitation in cancer care: patients' ability to prepare for major abdominal surgery. Scand J Caring Sci 2021(35):143-155.

5. Beck A, Vind Thaysen H, Hasselholt Soegaard C, Blaakaer J, Seibaek L. What matters to you? An investigation of patients' perspectives on and acceptability of prehabilitation in major cancer surgery. European Journal of Cancer Care 2021(30):1-10.

6. Brahmbhatt P, Sabiston CM, Lopez C, Chang E, Goodman J, Jones J, McCready D, R, all I, Rotstein S, Mina DS. Feasibility of Prehabilitation Prior to Breast Cancer Surgery: A Mixed-Methods Study. Frontiers in Oncology 2020(10).

7. Burke SM, Brunet J, Sabiston CM, Jack S, y, Grocott MPW, West MA. Patients' perceptions of quality of life during active treatment for locally advanced rectal cancer: the importance of preoperative exercise. Supportive Care in Cancer 2013(21):3345-3353.

8. Clode NJ, Perry MA, Wulff L. Does physiotherapy prehabilitation improve pre-surgical outcomes and influence patient expectations prior to knee and hip joint arthroplasty?. International Journal of Orthopaedic & Trauma Nursing 2018(30):14-19.

9. Collaço N, Henshall C, Belcher E, Canavan J, Merriman C, Mitchell J, Watson E. Patients' and healthcare professionals' views on a pre- and post-operative rehabilitation programme (SOLACE) for lung cancer: A qualitative study. J Clin Nurs 2022(31):283-293.

10. Cooper M, Chmelo J, Sinclair RCF, Charman S, Hallsworth K, Welford J, Phillips AW, Greystoke A, Avery L. Exploring factors influencing uptake and adherence to a home-based prehabilitation physical activity and exercise intervention for patients undergoing chemotherapy before major surgery (ChemoFit): a qualitative study. BMJ Open 2022(12):e062526-062526. doi: 10.1136/bmjopen-2022-062526.

11. Daun JT, Twomey R, Dort JC, Capozzi LC, Crump T, Francis GJ, Matthews TW, Chandarana SP, Hart RD, Schrag C, Matthews J, McKenzie CD, Lau H, Culos-Reed SN. A Qualitative Study of Patient and Healthcare Provider Perspectives on Building Multiphasic Exercise Prehabilitation into the Surgical Care Pathway for Head and Neck Cancer. Curr Oncol 2022(29):5942-5954. doi: 10.3390/curroncol29080469.

12. Ferreira V, Agnihotram RV, Bergdahl A, Rooijen SJV, Awasthi R, Carli F, Scheede-Bergdahl C. Maximizing patient adherence to prehabilitation: what do the patients say?. Supportive Care in Cancer 2018(26):2717-2723.

13. Finley DJ, Fay KA, Batsis JA, Stevens CJ, Sacks OA, Darabos C, Cook SB, Lyons KD. A feasibility study of an unsupervised, pre‐operative exercise program for adults with lung cancer. European Journal of Cancer Care 2020(29):1-10.

14. Gurunathan U, Tronstad O, Stonell C. Patient characteristics and preferences for a surgical prehabilitation program design: results from a pilot survey. J Cancer Res Clin Oncol 2022. doi: 10.1007/s00432-022-04420-4.

15. Karlsson E, Dahl O, Rydwik E, Nygren-Bonnier M, Bergenmar M. Older patients' attitudes towards, and perceptions of, preoperative physical activity and exercise prior to colorectal cancer surgery-a gap between awareness and action. Supportive care in cancer : official journal of the Multinational Association of Supportive Care in Cancer 2020(28):3945-3953.

16. Lam AKH, Fung OHY, Kwan C, Cheung JPY, Luk KDK, Chiu AYY, Descarreaux M, Szeto GPY, Wong AYL. The Concerns and Experiences of Patients With Lumbar Spinal Stenosis Regarding Prehabilitation and Recovery After Spine Surgery: A Qualitative Study. Arch Rehab Res Clin Trans 2022(4). doi: 10.1016/j.arrct.2022.100227.

17. Mooney M, Fitzsimons D, Richardson G. 'No more couch-potato!' Patients' experiences of a pre-operative programme of cardiac rehabilitation for those awaiting coronary artery bypass surgery. European Journal of Cardiovascular Nursing 2007(6):77-83.

18. Parker NH, Lee RE, O'Connor DP, Ngo-Huang A, Petzel MQB, Schadler K, Wang X, Xiao L, Fogelman D, Simpson R, Fleming JB, Lee JE, Tzeng CD, Sahai SK, Basen-Engquist K, Katz MHG. Supports and Barriers to Home-Based Physical Activity During Preoperative Treatment of Pancreatic Cancer: A Mixed-Methods Study. Journal of physical activity & health 2019(16):1113-1122.

19. Polen-De C, Langstraat C, Asiedu GB, Jatoi A, Kumar A. Advanced ovarian cancer patients identify opportunities for prehabilitation: A qualitative study. Gynecologic Oncology Reports 2021(36).

20. van der Zanden V, van der Zaag-Loonen HJ, Paarlberg KM, Meijer WJ, Mourits MJE, van Munster BJ. PREsurgery thoughts - thoughts on prehabilitation in oncologic gynecologic surgery, a qualitative template analysis in older adults and their healthcare professionals. Disabil Rehabil 2021:1-11.

21. Wang R, Yao C, Hung SH, Meyers L, Sutherland JM, Karimuddin A, Campbell KL, Conklin AI. Preparing for colorectal surgery: a qualitative study of experiences and preferences of patients in Western Canada. BMC Health Serv Res 2022(22):730-y. doi: 10.1186/s12913-022-08130-y.

22. Waterland JL, Ismail H, Amin B, Granger CL, Denehy L, Riedel B. Patient acceptance of prehabilitation for major surgery: an exploratory survey. Supportive care in cancer : official journal of the Multinational Association of Supportive Care in Cancer 2021(29):779-785.

23. Wu F, Laza-Cagigas R, Rampal T. Understanding Patients’ Experiences and Perspectives of Tele-Prehabilitation: A Qualitative Study to Inform Service Design and Delivery. Clin Pract 2022(12):640-652. doi: 10.3390/CLINPRACT12040067.

stylefix
