# Supplementary material for: What moves patients to participate in prehabilitation before major surgery? A mixed methods systematic review
Source: Int J Behav Nutr Phys Act. 2023 Jun 21;20:75. doi: 10.1186/s12966-023-01474-6 (PMC10286498; doi:10.1186/s12966-023-01474-6)
Supplement: Supplementary file 3 — Additional file 3: Methodological quality per study according to the MMAT-criteria [file 12966_2023_1474_MOESM3_ESM.docx]

**Additional file 2 Characteristics of included studies**

|  | **Type of study** | **Study aim** | **Study population** | **Sample size** | **Patient characteristics** | **Context and type of prehabilitation** | **Data collection** | **Data analysis** |
| --- | --- | --- | --- | --- | --- | --- | --- | --- |
| Agasi-Idenburg et al, 2020 [1] | Qualitative | To investigate the barriers, facilitators, and preferences for preoperative exercise programs in older patients scheduled for CRC surgery. | Frail patients aged 65 years and older, surgically treated for CRC. | n=15 | Mean age (SD) 72.7 (4.4), male 73%, | Physical activity or exercise prior to surgery. | In-depth interviews | Thematic content analysis |
| Banerjee et al, 2021 [2] | Qualitative | To explore patient perspectives of preoperative high intensity aerobic interval exercise before radical cystectomy. | Patients with bladder cancer listed for radical cystectomy. | n=14 | Mean age (SD) 72.3 (6.0), male 93% | Vigorous intensity aerobic interval exercise at an exercise facility prior to surgery. | Focus groups | Thematic (framework) analysis |
| Beck at al, 2020 [3] | Qualitative | To investigate the experiences, thoughts, and feelings that underlie and influence prehabilitation among cancer patients due to undergo major abdominal surgery. | Patients with CRC scheduled for CRS + HIPEC and patients with ovarian cancer scheduled for CRS. | n=16 | Median age 58, male 31% | Oral information and a leaflet with home-based, multimodal, preoperative recommendations. | Semi-structured interviews | Systematic text condensation |
| Beck et al, 2021 [4] | Mixed Methods | To investigate what patients with cancer, due to undergo major abdominal surgery, were able to do when provided with preoperative, home-based, multimodal recommendations presented in a leaflet. | Patients with CRC scheduled for CRS + HIPEC and patients with ovarian cancer scheduled for CRS. | Quantitative n=53 Qualitative n=5 | Colorectal cancer: mean age (SD) 66 (8.5), male 35%. Ovarian cancer: mean age (SD) 59.5 (12.4), female 100% | Leaflet with home-based, multimodal, preoperative recommendations. | Semi-structured interviews and analysis of patient notes | Descriptive statistics and systematic text condensation |
| Beck et al, 2021 [5] | Mixed Methods | To understand perspectives on and acceptability of prehabilitation among patients undergoing CRS with or without HIPEC. | Patients with CRC scheduled for CRS + HIPEC and patients with ovarian cancer scheduled for CRS. | Quantitative n=53 Qualitative n=31 | Mean age 62, male 27% | Leaflet with home-based, multimodal, preoperative recommendations. | Semi-structured interviews and analysis of patient notes | Descriptive statistics and systematic text condensation |
| Brahmbhatt et al, 2020 [6] | Mixed Methods | To assess the feasibility and acceptability of an individualized, home-based prehabilitation intervention prior to breast cancer surgery and to explore the potential benefit of prehabilitation on physical fitness and participant-reported physical and psychosocial well-being over time. | Patients diagnosed with stage I–III breast cancer undergoing surgery. | Quantitative n=22 Qualitative n=5 | Mean age (SD) 54.2 (11.0), female 100% | Individually tailored, home-based exercise prescriptions prior to surgery. | Semi-structured interviews | Descriptive statistics and thematic analysis |
| Burke et al, 2013 [7] | Qualitative | To explore advanced rectal cancer patients' experiences of quality of life during participation in a pre-surgery structured exercise program. | Patients diagnosed with locally advanced rectal cancer. | n=10 | Mean age (SD) 58.2 (7.7), male 30% | In-hospital supervised preoperative exercise intervention. | Semi-structured interviews | Phenomenological approach |
| Clode et al, 2018 [8] | Mixed Methods | To investigate the influence of prehabilitation on pre-operative pain and function and to explore the influence of prehabilitation on expectations, satisfaction and patient experience. | Patients awaiting elective total hip and total knee replacement due to osteoarthritis. | Quantitative n=75 Qualitative n=22 | *Prehabilitation:* mean age (SD) 67.6 (8.7), male 50%  *Usual care:* mean age (SD) 62.5 (12.6), male 52% | One-hour exercise and education sessions, twice weekly, for eight weeks. | Semi-structured interviews | Descriptive statistics and a general inductive method |
| Collaço et al, 2021 [9] | Qualitative | To explore patients’ and health care professionals’ views and experiences of a pre- and post-operative rehabilitation intervention. | Patients with early-stage lung cancer who underwent surgery for lung cancer. | n=17 | Age 40-49 6%, 50-59 0%, 60-69 24%, 70-79 53%, 80-89 17%, male 41% | Specialist lung cancer service to optimize health and fitness levels prior to and following lung cancer resections. | One-to-one telephone interviews | Thematic analysis |
| Cooper et al, 2022 [10] | Qualitative | To identify factors influencing uptake, engagement and adherence to a home-based physical activity and exercise intervention. | Patients with locally advanced operable oesophagogastric adenocarcinoma. | n=22 | Mean age (SD) 67.3 (8.2), male 82% | Home-based physical activity and exercise intervention (a combination of targeted daily step-based physical activity and strengthening exercises). | Focus groups and individual interviews | Thematic analysis |
| Daun et al, 2022 [11] | Qualitative | To understand patients’ and health care professionals’ perspectives on the role of multiphasic exercise prehabilitation. | Head and neck cancer surgical patients. | n=10 | Mean age (SD) 60.8 (8.5), male 90% | Multiphasic exercise prehabilitation across the surgical timeline. | Semi-structured interviews | Interpretive description methodology and constructivist philosophy. |
| Ferreira et al, 2018 [12] | Quantitative | To better understand patients’ perspectives of prehabilitation and to identify factors related to program adherence. | Patients awaiting colorectal or lung cancer surgery. | n=52 | Mean age (SD) 66.9 (12.1), male 54% | Multimodal prehabilitation program | Questionnaire taken face-to-face or over telephone | Descriptive statistics |
| Finley et al, 2020 [13] | Mixed Methods | To explore the feasibility, acceptability and perceived utility of the provision of a wearable fitness device and an exercise pre- scription from a surgeon, prior to surgery for lung cancer. | Patients scheduled for lung cancer surgery. | n=28 | Mean age (SD) 67.3 (10.6), male 43% | Exercise prescription from a surgeon and use of a smartwatch. | Semi-structured telephone interviews | Descriptive statistics and content analysis |
| Gurunathan et al, 2022 [14] | Quantitative | To gain insight into the perception of patients about prehabilitation and to explore the preferences and barriers which will inform the design of a patient-centred prehabilitation program. | Adult patients undergoing elective major abdominal surgery | n=24 | Mean age (SD) 69 (14), male 58% | Multimodal prehabilitation program | Survey | Descriptive statistics |
| Karlsson et al, 2020 [15] | Qualitative | To describe older people’s attitudes and perceptions towards physical activity and exercise when scheduled for colorectal cancer surgery. | Patients aged 70 years and older scheduled for colorectal cancer surgery. | n=17 | Median age (range) 75 (70-91), male 53% | Physical activity and exercise. | Semi-structured interviews | Descriptive statistics and content analysis |
| Lam et al, 2022 [16] | Qualitative | To understand patients’ concerns and considerations before LSS surgery, their perspectives toward prehabilitation, and experiences after LSS surgery. | Patients aged 50 years or older diagnosed with LSS, 6 months after posterior open decompression surgery. | n=25 | *Prehabilitation:* mean age (SD) 66.4 (4.4), male 67%  *Usual care:* mean age (SD) 66.5 (8.0), male 67% | A 6-week structured prehabilitation program vs usual pre-operative care. | Face-to-face interviews | Thematic analysis |
| Mooney et al, 2007 [17] | Qualitative | To describe patients' experience of a pre-operative program of cardiac rehabilitation. | Patients awaiting coronary artery bypass surgery. | n=7 | Age 54-74 | A 12-week cardiac prehabilitation program | Unstructured interviews | Analysis using Colaizzi's framework |
| Parker et al, 2019 [18] | Mixed Methods | To characterize relationships between socioecological factors and physical activity among patients enrolled in a home-based exercise program concurrent with preoperative treatment for pancreatic cancer. | Patients diagnosed with locally advanced rectal cancer. | Quantitative n=50 Qualitative n=10 | Mean age (SD) 66 (8), male 52% | Home-based, multimodal exercise program. | Semi-structured interviews | Descriptive statistics and qualitative analysis |
| Polen-De et al, 2021 [19] | Qualitative | To understand and evaluate how patients with advanced ovarian cancer undergoing NACT view exercise and physical activity during treatment. | Patients with advanced ovarian cancer who had undergone NACT. | n=15 | Mean age 64.3, female 100% | Exercise and physical activity during NACT | Semi-structured telephone interviews | Qualitative analysis |
| van der Zanden et al, 2021 [20] | Qualitative | To investigate opinions, thoughts, and desires of elderly gynecological oncological patients and health care professionals with regard to feasibility, content and indications for prehabilitation, and what potential barriers might exist. | Patients aged 60 years and older scheduled for, or underwent, oncological gynecological surgery. | n=16 | Median age (range) 70 (62-85), female 100% | Not specified | Semi-structured interviews | Thematic analysis |
| Wang et al, 2022 [21] | Qualitative | To describe experiences and explore preferences for multimodal prehabilitation among patients waiting for complex colorectal surgery. | Patients who received abdominal surgery for colorectal conditions. | n=19 | Median age (range) 58 (31-72), male 37% | Multimodal prehabilitation | Focus groups | Thematic analysis |
| Waterland et al, 2021 [22] | Quantitative | To assess the acceptability of prehabilitation among patients who scheduled for major gastrointestinal and urological cancer surgery. To assess the current levels of daily exercise in patients eligible for prehabilitation and identify potential barriers and enablers to commencing, continuing and completing prehabilitation. | Patients scheduled for major gastrointestinal and urological cancer surgery. | n=103 | Median age (IQR) 61 (52-70), male 47% | Not specified | Survey | Descriptive statistics |
| Wu et al, 2022 [23] | Qualitative | To explore patients’ experiences and perspectives of tele-prehabilitation. | Adult surgery patients who participated in a tele-prehabilitation program. | n = 22 | Median age (range) 66 (42-83), male 50% | Structured teleprehabilitation program | Semi-structured interviews | Thematic analysis |

**References**

1. Agasi-Idenburg CS, Zuilen MK, Westerman MJ, Punt CJA, Aaronson NK, Stuiver MM. I am busy surviving - Views about physical exercise in older adults scheduled for colorectal cancer surgery. Journal of geriatric oncology 2020(11):444-450.

2. Banerjee S, Semper K, Skarparis K, Naisby J, Lewis L, Cucato G, Mills R, Rochester M, Saxton J. Patient perspectives of vigorous intensity aerobic interval exercise prehabilitation prior to radical cystectomy: a qualitative focus group study. Disabil Rehabil 2021(43):1084-1091.

3. Beck A, Thaysen HV, Soegaard CH, Blaakaer J, Seibaek L. Investigating the experiences, thoughts, and feelings underlying and influencing prehabilitation among cancer patients: a qualitative perspective on the what, when, where, who, and why. Disabil Rehabil 2020:1-8.

4. Beck A, Thaysen HV, Soegaard CH, Blaakaer J, Seibaek L. Prehabilitation in cancer care: patients' ability to prepare for major abdominal surgery. Scand J Caring Sci 2021(35):143-155.

5. Beck A, Vind Thaysen H, Hasselholt Soegaard C, Blaakaer J, Seibaek L. What matters to you? An investigation of patients' perspectives on and acceptability of prehabilitation in major cancer surgery. European Journal of Cancer Care 2021(30):1-10.

6. Brahmbhatt P, Sabiston CM, Lopez C, Chang E, Goodman J, Jones J, McCready D, R, all I, Rotstein S, Mina DS. Feasibility of Prehabilitation Prior to Breast Cancer Surgery: A Mixed-Methods Study. Frontiers in Oncology 2020(10).

7. Burke SM, Brunet J, Sabiston CM, Jack S, y, Grocott MPW, West MA. Patients' perceptions of quality of life during active treatment for locally advanced rectal cancer: the importance of preoperative exercise. Supportive Care in Cancer 2013(21):3345-3353.

8. Clode NJ, Perry MA, Wulff L. Does physiotherapy prehabilitation improve pre-surgical outcomes and influence patient expectations prior to knee and hip joint arthroplasty?. International Journal of Orthopaedic & Trauma Nursing 2018(30):14-19.

9. Collaço N, Henshall C, Belcher E, Canavan J, Merriman C, Mitchell J, Watson E. Patients' and healthcare professionals' views on a pre- and post-operative rehabilitation programme (SOLACE) for lung cancer: A qualitative study. J Clin Nurs 2022(31):283-293.

10. Cooper M, Chmelo J, Sinclair RCF, Charman S, Hallsworth K, Welford J, Phillips AW, Greystoke A, Avery L. Exploring factors influencing uptake and adherence to a home-based prehabilitation physical activity and exercise intervention for patients undergoing chemotherapy before major surgery (ChemoFit): a qualitative study. BMJ Open 2022(12):e062526-062526. doi: 10.1136/bmjopen-2022-062526.

11. Daun JT, Twomey R, Dort JC, Capozzi LC, Crump T, Francis GJ, Matthews TW, Chandarana SP, Hart RD, Schrag C, Matthews J, McKenzie CD, Lau H, Culos-Reed SN. A Qualitative Study of Patient and Healthcare Provider Perspectives on Building Multiphasic Exercise Prehabilitation into the Surgical Care Pathway for Head and Neck Cancer. Curr Oncol 2022(29):5942-5954. doi: 10.3390/curroncol29080469.

12. Ferreira V, Agnihotram RV, Bergdahl A, Rooijen SJV, Awasthi R, Carli F, Scheede-Bergdahl C. Maximizing patient adherence to prehabilitation: what do the patients say?. Supportive Care in Cancer 2018(26):2717-2723.

13. Finley DJ, Fay KA, Batsis JA, Stevens CJ, Sacks OA, Darabos C, Cook SB, Lyons KD. A feasibility study of an unsupervised, pre‐operative exercise program for adults with lung cancer. European Journal of Cancer Care 2020(29):1-10.

14. Gurunathan U, Tronstad O, Stonell C. Patient characteristics and preferences for a surgical prehabilitation program design: results from a pilot survey. J Cancer Res Clin Oncol 2022. doi: 10.1007/s00432-022-04420-4.

15. Karlsson E, Dahl O, Rydwik E, Nygren-Bonnier M, Bergenmar M. Older patients' attitudes towards, and perceptions of, preoperative physical activity and exercise prior to colorectal cancer surgery-a gap between awareness and action. Supportive care in cancer : official journal of the Multinational Association of Supportive Care in Cancer 2020(28):3945-3953.

16. Lam AKH, Fung OHY, Kwan C, Cheung JPY, Luk KDK, Chiu AYY, Descarreaux M, Szeto GPY, Wong AYL. The Concerns and Experiences of Patients With Lumbar Spinal Stenosis Regarding Prehabilitation and Recovery After Spine Surgery: A Qualitative Study. Arch Rehab Res Clin Trans 2022(4). doi: 10.1016/j.arrct.2022.100227.

17. Mooney M, Fitzsimons D, Richardson G. 'No more couch-potato!' Patients' experiences of a pre-operative programme of cardiac rehabilitation for those awaiting coronary artery bypass surgery. European Journal of Cardiovascular Nursing 2007(6):77-83.

18. Parker NH, Lee RE, O'Connor DP, Ngo-Huang A, Petzel MQB, Schadler K, Wang X, Xiao L, Fogelman D, Simpson R, Fleming JB, Lee JE, Tzeng CD, Sahai SK, Basen-Engquist K, Katz MHG. Supports and Barriers to Home-Based Physical Activity During Preoperative Treatment of Pancreatic Cancer: A Mixed-Methods Study. Journal of physical activity & health 2019(16):1113-1122.

19. Polen-De C, Langstraat C, Asiedu GB, Jatoi A, Kumar A. Advanced ovarian cancer patients identify opportunities for prehabilitation: A qualitative study. Gynecologic Oncology Reports 2021(36).

20. van der Zanden V, van der Zaag-Loonen HJ, Paarlberg KM, Meijer WJ, Mourits MJE, van Munster BJ. PREsurgery thoughts - thoughts on prehabilitation in oncologic gynecologic surgery, a qualitative template analysis in older adults and their healthcare professionals. Disabil Rehabil 2021:1-11.

21. Wang R, Yao C, Hung SH, Meyers L, Sutherland JM, Karimuddin A, Campbell KL, Conklin AI. Preparing for colorectal surgery: a qualitative study of experiences and preferences of patients in Western Canada. BMC Health Serv Res 2022(22):730-y. doi: 10.1186/s12913-022-08130-y.

22. Waterland JL, Ismail H, Amin B, Granger CL, Denehy L, Riedel B. Patient acceptance of prehabilitation for major surgery: an exploratory survey. Supportive care in cancer : official journal of the Multinational Association of Supportive Care in Cancer 2021(29):779-785.

23. Wu F, Laza-Cagigas R, Rampal T. Understanding Patients’ Experiences and Perspectives of Tele-Prehabilitation: A Qualitative Study to Inform Service Design and Delivery. Clin Pract 2022(12):640-652. doi: 10.3390/CLINPRACT12040067.

stylefix
